# Probiotic Intervention With *Bacillus coagulans* and *Lactobacillus acidophilus* in Small Animals Experimental Ulcerative Colitis: Insights From a Small Animal Model

**DOI:** 10.1002/vms3.70883

**Published:** 2026-09-29

**Authors:** Fatemeh Shayesteh, Milad Shahini Shams Abadi, Leila Rouhi, Abbas Doosti, Nader Bagheri

**Affiliations:** ^1^ Biotechnology Research Center, ShK.C. Islamic Azad University Shahrekord Iran; ^2^ Clinical Biochemistry Research Center, Basic Health Sciences Institute Shahrekord University of Medical Sciences Shahrekord Iran; ^3^ Department of Biology, ShK.C. Islamic Azad University Shahrekord Iran; ^4^ Cellular and Molecular Research Center, Basic Health Sciences Institute Shahrekord University of Medical Sciences Shahrekord Iran

**Keywords:** *Bacillus coagulans*, inflammasome, *Lactobacillus acidophilus*, probiotics, ulcerative colitis, veterinary gastroenterology

## Abstract

Inflammatory bowel disease is a significant health concern not only in humans but also in companion animals and livestock, where it manifests as chronic diarrhoea, abdominal pain, and mucosal injury. Probiotics have gained increasing attention as potential therapeutic agents due to their immunomodulatory effects and ability to restore intestinal homeostasis. This study investigated the therapeutic efficacy of *Bacillus coagulans* and *Lactobacillus acidophilus* in an acetic acid–induced ulcerative colitis model in Wistar rats. Forty male rats were randomly assigned to control, colitis, mesalazine, *B. coagulans*, and *L. acidophilus* treatment groups. Disease severity was evaluated by macroscopic and histopathological scoring, colon length and weight, pro‐inflammatory cytokine levels (IL‐1β, IL‐17), and inflammasome‐associated gene expression (NLRP3, Caspase‐1, IL‐18, TNF‐α). The results showed that colitis induction significantly increased pro‐inflammatory cytokine expression and histological damage compared with controls. Both probiotics markedly reduced inflammatory markers, improved colon morphology, and attenuated inflammasome activation. Their protective effects were comparable to mesalazine, the standard treatment. These findings suggest that *B. coagulans* and *L. acidophilus* can serve as effective adjunct therapies for ulcerative colitis in veterinary practice, with potential translational applications for companion animals and livestock.

## Graphical abstract

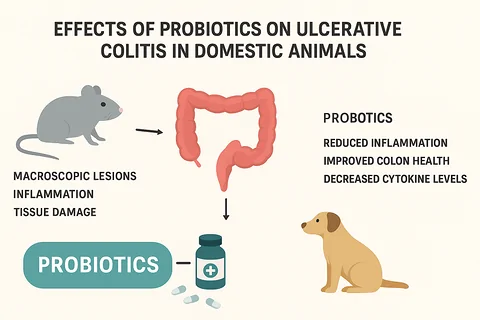

## 1 Introduction

Inflammatory bowel diseases are not limited to humans; they are increasingly recognized in veterinary medicine, particularly in companion animals, such as dogs and cats, as well as in livestock species (Allenspach 2015). Ulcerative colitis and colitis‐like syndromes in animals are characterized by chronic diarrhoea, weight loss, abdominal discomfort, and impaired nutrient absorption (Goyal et al. 2014). Although their pathogenesis remains complex, evidence suggests that dysregulated host–microbiota interactions and inappropriate immune responses play pivotal roles (Washabau and Day 2013). Current therapeutic measures rely heavily on corticosteroids, antibiotics, and immunomodulators, but treatment failures and side effects are common (Dandrieux 2016). These limitations have stimulated the search for alternative, safer approaches. Probiotics have emerged as promising candidates in veterinary gastroenterology due to their immunomodulatory and microbiota‐restoring effects (Schmitz and Suchodolski 2016).

Animal models are essential for studying intestinal inflammation and evaluating new therapeutic strategies. In veterinary research, murine models, such as dextran sulphate sodium–induced colitis, serve as accessible platforms to investigate pathophysiology before translation to clinical veterinary cases (Wirtz et al. 2017). These models reproduce histopathological features of colitis observed in dogs, cats, and cattle, including mucosal ulceration, epithelial barrier dysfunction, and infiltration of inflammatory cells (Jergens and Simpson 2012). Thus, small animal experiments provide both mechanistic insights and translational value for managing intestinal inflammation in domestic species.

One of the central mechanisms in the pathogenesis of colitis across species is inflammasome signalling. Inflammasomes are cytosolic protein complexes that sense microbial or endogenous danger signals and activate caspase‐1, leading to the maturation of interleukin‐1β (IL‐1β) and interleukin‐18 (IL‐18) (Lamkanfi and Dixit 2014; Man et al. 2017). The aberrant activation of inflammasomes, particularly the NLRP3 inflammasome, has been associated with persistent mucosal inflammation and colonic tissue damage in both human and veterinary cases (Zhen and Zhang 2019). Targeting inflammasome pathways could therefore offer therapeutic opportunities not only for people but also for dogs, cats, and livestock suffering from colitis.

Probiotics are defined as live microorganisms that, when administered in adequate amounts, confer health benefits to the host (Hill et al. 2014; Ekhteraei‐Tousi et al. 2015). In veterinary species, probiotics have been shown to reduce diarrhoea, modulate immune responses, and improve growth performance (Uyeno et al. 2015). Mechanistically, probiotics reinforce epithelial integrity, compete with pathogenic bacteria, produce antimicrobial compounds, and regulate immune cell activity (Bron et al. 2017a, 2017b). More recently, their role in modulating inflammasome activation has been highlighted, providing a new perspective on their benefits in inflammatory bowel conditions of animals (Li et al. 2021; Piri‐Gharaghie et al. 2024).

*Bacillus coagulans* is a spore‐forming lactic acid bacterium with unique resilience to harsh environmental and gastrointestinal conditions (Adimpong et al. 2012). This trait is particularly relevant for veterinary applications, as probiotics must withstand variable storage conditions and digestive environments in farm animals. Studies have reported its positive effects on gut health in poultry, pigs, and companion animals, where it reduced diarrhoea, enhanced nutrient absorption, and modulated immunity (Goyal et al. 2014; Hun 2020a). In murine colitis models, *B. coagulans* supplementation decreased inflammation and improved colonic histopathology (Majeed et al. 2016). Such evidence highlights its potential application in veterinary colitis management.

*Lactobacillus acidophilus* is another probiotic widely used in veterinary medicine due to its strong capacity to colonize the intestinal tract and exert antimicrobial and immunomodulatory effects (Ouwehand, Salminen, and Isolauri 2002, Hernández‐González et al. 2021). Supplementation in dogs and calves has been associated with improved gut health and reduced gastrointestinal infections (Schmitz and Suchodolski 2016). In experimental models of colitis, *L. acidophilus* reduces inflammatory cytokine expression, enhances epithelial barrier function, and modulates inflammasome activity (Wang et al. 2018). These properties make it highly relevant for both companion animal gastroenterology and farm animal production systems.

Combination therapies using multiple probiotic strains may provide broader benefits than single‐strain supplementation (Chapman et al. 2011a; Ekhteraei‐Tousi et al. 2015). Veterinary probiotic formulations often include blends of *Bacillus* and *Lactobacillus* species to target different aspects of gut health (Timmerman et al. 2004; Moosavi‐Kohnehsari et al. 2025). The combination of *B. coagulans* and *L. acidophilus* may synergistically regulate microbiota composition, enhance mucosal integrity, and modulate immune signalling pathways. Despite promising preliminary results, few studies have specifically evaluated their combined effects on inflammasome signalling in animal colitis models.

Investigating how probiotics influence inflammasome regulation in colitis is essential for veterinary applications. The overactivation of NLRP3 inflammasome has been linked to chronic diarrhoea and poor mucosal healing in dogs and cats with inflammatory bowel disease (Allenspach 2015). Probiotics that suppress inflammasome overactivation could therefore represent novel adjunct therapies. *Bacillus coagulans* and *L. acidophilus* have demonstrated the capacity to influence Toll‐like receptor and cytokine pathways, suggesting potential indirect regulation of inflammasome activity (Bermudez‐Brito et al. 2012).

Colitis in veterinary species has significant welfare and economic implications. Chronic inflammatory gut diseases reduce productivity in farm animals, impair growth in young stock, and increase morbidity in pets (Esmatabadi et al. 2018). Safe, affordable, and effective probiotic interventions could improve animal health outcomes, reduce reliance on antibiotics, and support sustainable livestock production (Uyeno et al. 2015; Leistikow et al. 2022). Small animal experimental models therefore sere as a valuable bridge, linking basic mechanistic studies with veterinary clinical practice.

In this context, the present study evaluates the probiotic intervention of *B. coagulans* and *L. acidophilus* in a murine model of ulcerative colitis, with emphasis on inflammasome pathway gene expression. The objective is to explore their mechanistic role in reducing intestinal inflammation and to provide translational insights for veterinary medicine. The findings are expected to advance the understanding of probiotic applications in veterinary colitis, contribute to safer therapeutic alternatives, and promote improved health and welfare in small and large animals.

## 2 Materials and Methods

### 2.1 Materials

Ketamine hydrochloride (Alfasan) and xylazine hydrochloride (Alfasan) were used for anaesthesia. Acetic acid (Merck) and phosphate‐buffered saline (PBS; pH 7.4; Gibco) were used for colitis induction. Culture media, including de Man, Rogosa and Sharpe (MRS) agar and tryptic soy broth (TSB), were purchased from Merck (Germany). Probiotic strains *B. coagulans* (IBRC‐M 10791) and *L. acidophilus* (PTCC 1643) were obtained from the Iranian Biological Resource Center (Tehran, Iran) and the Persian Type Culture Collection (Tehran, Iran), respectively. Mesalazine was purchased from Sigma‐Aldrich. The RNA extraction reagent RNX‐Plus was obtained from SinaClone, and the cDNA synthesis kit (YT4500) was obtained from Yekta Tajhiz Azma. SYBR Green Master Mix (RR820Q) was purchased from Takara (Shiga, Japan). ELISA kits for IL‐17 and IL‐1β were supplied by Karmania Parsgene (Kerman, Iran). All other chemicals and reagents used were of analytical grade.

### 2.2 Experimental Animals and Grouping

A total of 40 male Wistar rats (240–250 g) were obtained from the Pasteur Institute (Tehran, Iran). Animals were housed under standard laboratory conditions (22–25°C, 12‐h light/dark cycle) with ad libitum access to food and water. After a 1‐week acclimatization period, rats were randomly assigned into 5 groups (*n* = 8 per group): (1) Control, (2) Ulcerative colitis, (3) mesalazine‐treated, (4) *B. coagulans* (IBRC‐M 10791), and (5) *L. acidophilus* (PTCC 1643) (Table 1).

**TABLE 1:** Experimental design showing the grouping of animals, induction of colitis, and treatment regimen administered by intrarectal injection and daily oral gavage.

Experimental group	Single dose intrarectal administration	Daily oral gavage
Control	100 µL of 9% normal saline	—
Ulcerative colitis	100 µL of 4% acetic acid	—
Mesalazine	100 µL of 4% acetic acid	300 mg/kg^a^
*Bacillus coagulans*	100 µL of 4% acetic acid	10^9^ CFU/mL
*Lactobacillus acidophilus*	100 µL of 4% acetic acid	10^9^ CFU/mL

^a^ Daily intravenous injection.

### 2.3 Induction of Experimental Colitis

Following 24 h of fasting (water allowed ad libitum), colitis was induced by intrarectal administration of 100 µL 4% acetic acid under ketamine (50 mg/kg) and xylazine (10 mg/kg) anaesthesia. To prevent reflux, rats were maintained in a head‐down position for 40 s, and the rectal lumen was flushed with PBS (pH 7.4). Control animals received 100 µL of PBS only. Colitis was confirmed after 24 h.

### 2.4 Treatment Protocol

Following colitis induction, the intervention groups received daily oral gavage for 7 consecutive days as follows: *B. coagulans* suspension (10^9^ CFU/mL) and *L. acidophilus* suspension (10^9^ CFU/mL). The positive control group received mesalazine (300 mg/kg/day, oral). All suspensions were freshly prepared. From day 8 to 10, animals were maintained on plain water. On day 10, rats were deeply anesthetized and euthanized; colonic tissues were collected for further analyses.

### 2.5 Preparation of Probiotic Suspensions

Colonies of *B. coagulans* and *L. acidophilus* were cultured on MRS agar and subsequently transferred into TSB broth until reaching an optical density corresponding to ∼10^9^ CFU/mL. Cultures were centrifuged (10,000 rpm, 20 min), and bacterial pellets were resuspended in sterile saline. The final bacterial concentration was adjusted against the 0.5 McFarland standard.

### 2.6 Macroscopic Evaluation of Colonic Damage

Macroscopic colonic injury was evaluated using the Wallace and Keenan scoring system, which considers hyperaemia, ulceration, and the extent of inflammation (scores 0–5). Each colon was scored independently by two blinded observers, and cumulative scores were used to quantify lesion severity.

### 2.7 Quantitative Real‐Time PCR

Total RNA was extracted from frozen colonic samples using RNX‐Plus solution (SinaClone, Iran). RNA concentration and purity were determined spectrophotometrically (Nanodrop). cDNA synthesis was performed using a commercial kit (YT4500, Yekta Tajhiz Azma, Iran). Quantitative real‐time PCR (qPCR) amplification was carried out with SYBR Green Master Mix (Takara) on a Rotor‐Gene RG‐300 system. The relative mRNA expression of inflammasome‐associated genes (IL‐18, NLRP3, Caspase‐1, TNF‐α) was normalized against β‐actin using the 2^−^^ΔΔCt^ method. Primer sequences are presented in Table 2.

**TABLE 2:** Primer sequences used for quantitative real‐time PCR analysis of target genes in colonic tissues.

Gene	Product length (bp)	Temperature (°C)	Gene accession number	Primer (5'‐3')
β‐Actin	173	60	NM_031144	F: GCCCGTGAAAAGATGAC R: AGCGCGTAACCCTCATAGAT
TNF‐α	152	63	HQ201305	F: CTGGCGTGTTCATCCGTTC R: GGCTCTGAGGAGTAGACGAT
Caspase‐1	142	60	NM_012762	F: AGACTACAGATGCCAACCACT R: CATTTTGGGGATTATTGGCTTC
IL‐18	106	60	NM_019165	F: GGTCTGATTCCAAGTCTCCAT R: GCTGCCATACCAGAAGAAGG
NLRP3	164	60	NM_001191642	F: CTCACCTCACACTCCTGCTG R: TCACCCAACTGTAGGCTCTGC

### 2.8 Histopathological Assessment

For histological analysis, colonic tissues were fixed in 10% neutral‐buffered formalin, dehydrated in graded ethanol, embedded in paraffin, and sectioned at 4 µm. Haematoxylin–eosin (H&E) staining was performed, and slides were evaluated at 100× magnification. Histopathological damage was graded (Bron et al. 2017a, 2017b) based on crypt damage, mucosal and submucosal involvement, inflammatory cell infiltration, and vascular density, according to Dieleman's criteria (Table 3).

**TABLE 3:** Scoring system used for histopathological evaluation of colitis severity based on crypt damage, depth of tissue involvement, inflammatory response, and percentage of tissue affected.

Score	Degree of crypt damage	Depth of tissue damage	Degree of inflammation	Percentage of tissue involvement
0	Intact epithelium and crypts	No damage	No inflammation	0
1	Damage to 1/3 of crypts	Mucosal damage	Mild	1–25
2	Damage to 2/3 of crypts	Mucosal and submucosal damage	Moderate	26–50
3	Crypts destroyed, surface epithelium present	Transmural damage	Severe	51–75
4	Crypts destroyed, surface epithelium absent	Transmural damage	Severe	76–100

### 2.9 Determination of Pro‐Inflammatory Cytokines

Colonic tissues were homogenized in RIPA buffer, and protein extracts were obtained by centrifugation (13,000 rpm, 20 min, 4°C). Total protein content was quantified using the Bradford assay. Concentrations of IL‐17 and IL‐1β were determined using commercial ELISA kits (Karmania Parsgene) with a sensitivity of 8 pg/mL, according to the manufacturer's protocols.

### 2.10 Statistical Analysis

All data were expressed as mean ± standard error of the mean (SEM). Statistical comparisons among the experimental groups were performed using one‐way analysis of variance followed by Tukey's post hoc test for multiple comparisons. Non‐parametric data, such as histopathological scores, were analyzed using the Kruskal–Wallis test followed by Dunn's post hoc test. A value of *p* < 0.05 was considered statistically significant. All analyses were carried out using GraphPad Prism version 9.0 (GraphPad Software).

## 3 Results

### 3.1 Relative Gene Expression of Inflammasome‐Related Cytokines in Colonic Tissue

The relative expression levels of IL‐18, NLRP3, Caspase‐1, and TNF‐α were analyzed in colonic tissues of experimental groups. The induction of colitis resulted in a significant upregulation of IL‐18 (Fold Change [FC] = –7.56, *p* = 0.0004), NLRP3 (FC = –11.22, *p* = 0.0001), Caspase‐1 (FC = –12.31, *p* < 0.0001), and TNF‐α (FC = –6.22, *p* < 0.0001) compared with the healthy control group. The administration of mesalazine (300 mg/kg) markedly reduced the expression of IL‐18 (FC = 7.90, *p* = 0.0002), NLRP3 (FC = 11.99, *p* < 0.0001), Caspase‐1 (FC = 11.64, *p* < 0.0001), and TNF‐α (FC = 5.94, *p* < 0.0001) compared with the colitis group. Similarly, treatment with *L. acidophilus* significantly downregulated IL‐18 (FC = 7.80, *p* = 0.0004), NLRP3 (FC = 9.09, *p* = 0.0028), Caspase‐1 (FC = 12.26, *p* = 0.0001), and TNF‐α (FC = 7.34, *p* = 0.0001). In the *B. coagulans* group, IL‐18 (FC = 5.77, *p* = 0.0255), NLRP3 (FC = 10.02, *p* = 0.0015), Caspase‐1 (FC = 10.08, *p* = 0.0006), and TNF‐α (FC = 7.35, *p* < 0.0001) expression were also significantly reduced compared with the colitis group. These findings indicate that both probiotics exert beneficial effects on the modulation of inflammasome‐related gene expression in experimental ulcerative colitis (Figure 1).

**FIGURE 1:**
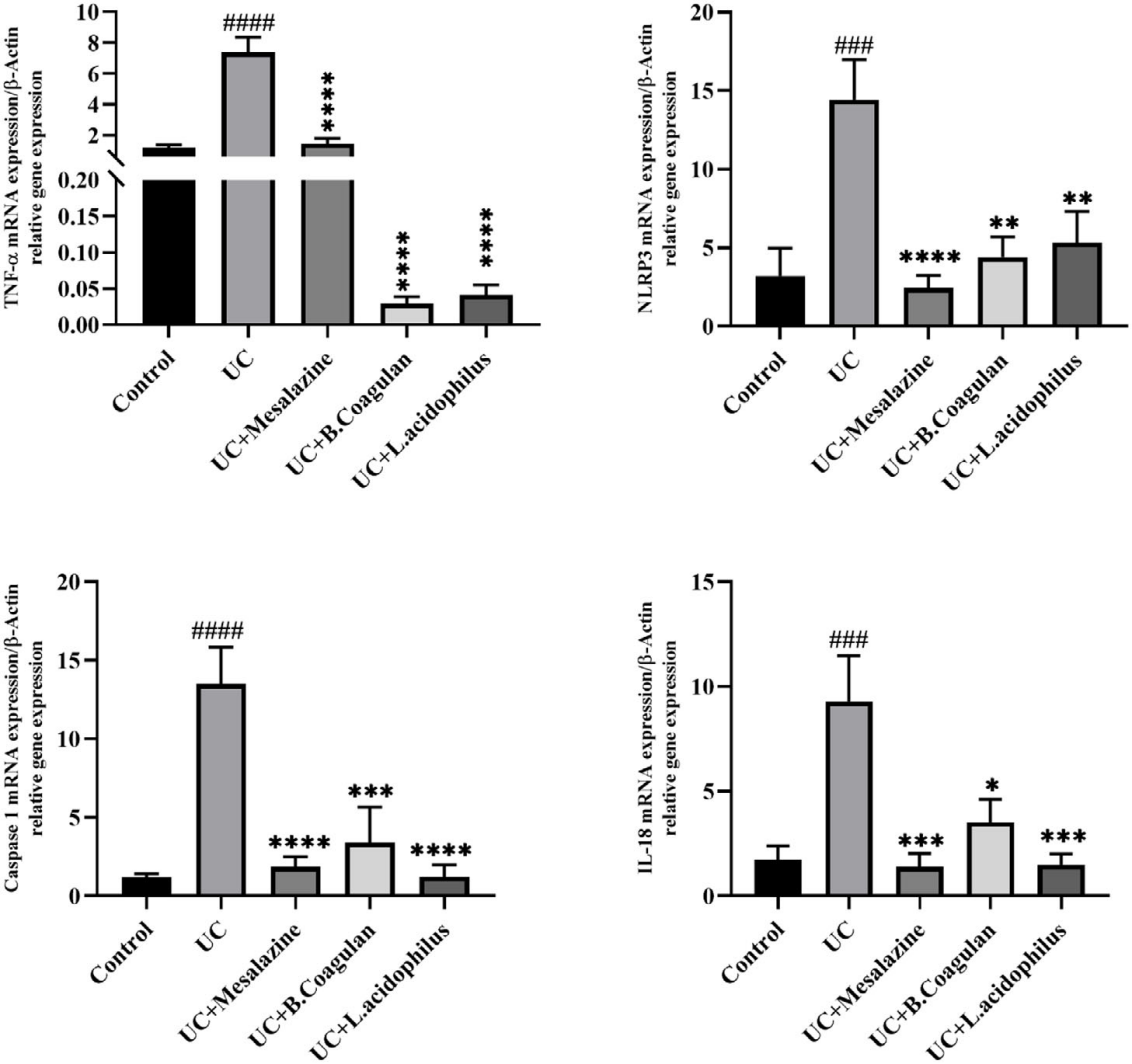
Effects of mesalazine and probiotics (Lactobacillus acidophilus and Bacillus coagulans) on the expression of IL‐18, TNF‐α, NLRP3, and Caspase‐1 genes in acetic acid–induced ulcerative colitis. mRNA expression in colonic tissue was determined by qPCR. Data are presented as mean ± SEM (n = 8 per group). Statistical significance: *p < 0.05, **p < 0.01, ***p < 0.001 and ****p < 0.0001 compared with the ulcerative colitis group.

### 3.2 Results of Tissue Protein Analysis of IL‐17 and IL‐1β in Colonic Tissue

Our findings demonstrated that the induction of ulcerative colitis markedly increased the tissue protein levels of IL‐1β (FC = −208.5, *p* < 0.0001) and IL‐17 (FC = −69.51, *p* < 0.0001) in colonic tissue compared with the healthy control group. Treatment with mesalazine (300 mg/kg, standard therapy) significantly reduced the levels of IL‐1β (FC = 0.146, *p* < 0.0001) and IL‐17 (FC = 49.70, *p* < 0.0001) compared with the colitis group. Similarly, the administration of probiotic strains resulted in a significant reduction in both cytokines: *L. acidophilus* (IL‐1β: FC = 5.214, *p* < 0.0001; IL‐17: FC = 1.139, *p* < 0.0001) and *B. coagulans* (IL‐1β: FC = 0.207, *p* < 0.0001; IL‐17: FC = 1.134, *p* < 0.0001). These results indicate that both individual and combined probiotic interventions attenuated the elevated tissue protein levels of IL‐1β and IL‐17 in colitis, comparable to the standard mesalazine treatment (Figure 2).

**FIGURE 2:**
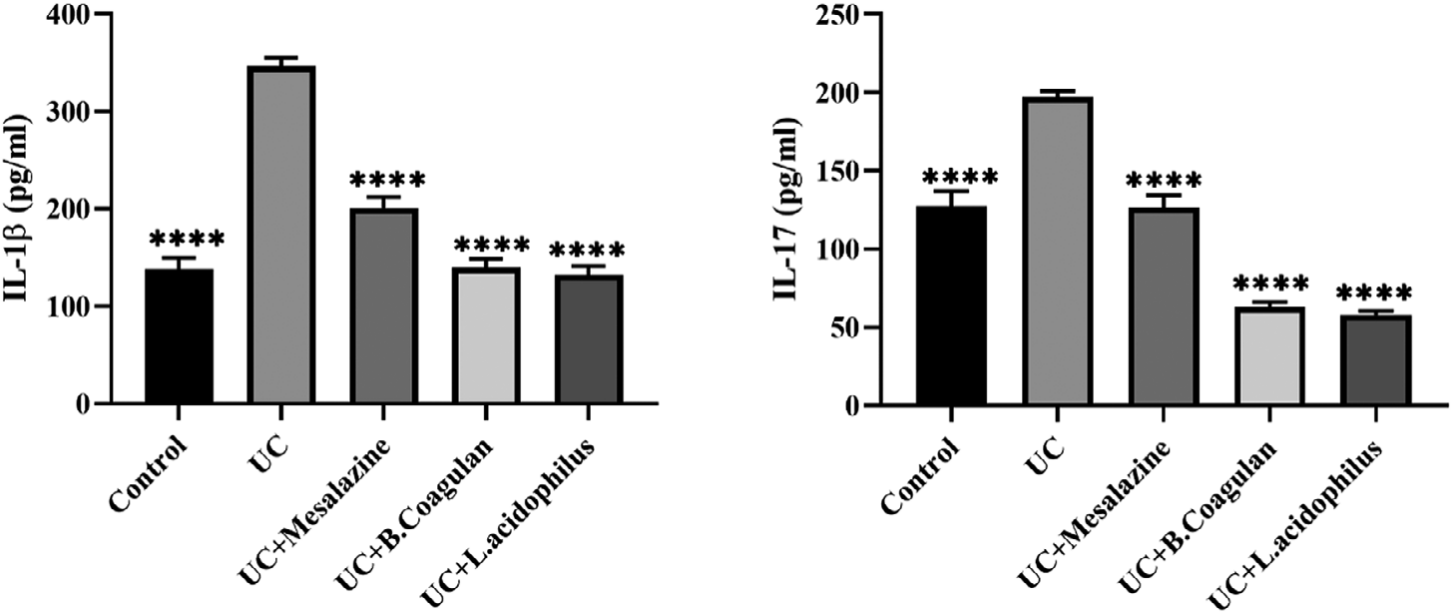
Effects of Lactobacillus acidophilus, Bacillus coagulans, and mesalazine on tissue protein levels of IL‐17 and IL‐1β in acetic acid–induced ulcerative colitis. Protein concentrations were measured in colonic tissue using ELISA. Data are presented as mean ± SEM. ****p < 0.0001 vs. ulcerative colitis group. Number of mice per group = 8.

### 3.3 Macroscopic Evaluation of Colonic Tissue in Experimental Groups

Macroscopic assessment revealed that treatment with *B. coagulans* and *L. acidophilus* markedly improved colonic lesions in acetic acid–induced ulcerative colitis (Figure 3). As illustrated in Figure 3b, the administration of acetic acid caused severe colonic damage characterized by ulceration, hyperaemia, inflammation, and oedema. In contrast, Figure 3c–e demonstrates a clear reduction in inflammation and ulceration in the colonic tissue of groups treated with *B. coagulans*, *L. acidophilus*, and mesalazine. Based on the scoring system described in Section 2, the therapeutic efficacy of each intervention in ameliorating macroscopic colonic damage was quantified, and the results are summarized in Table 3.

**FIGURE 3:**
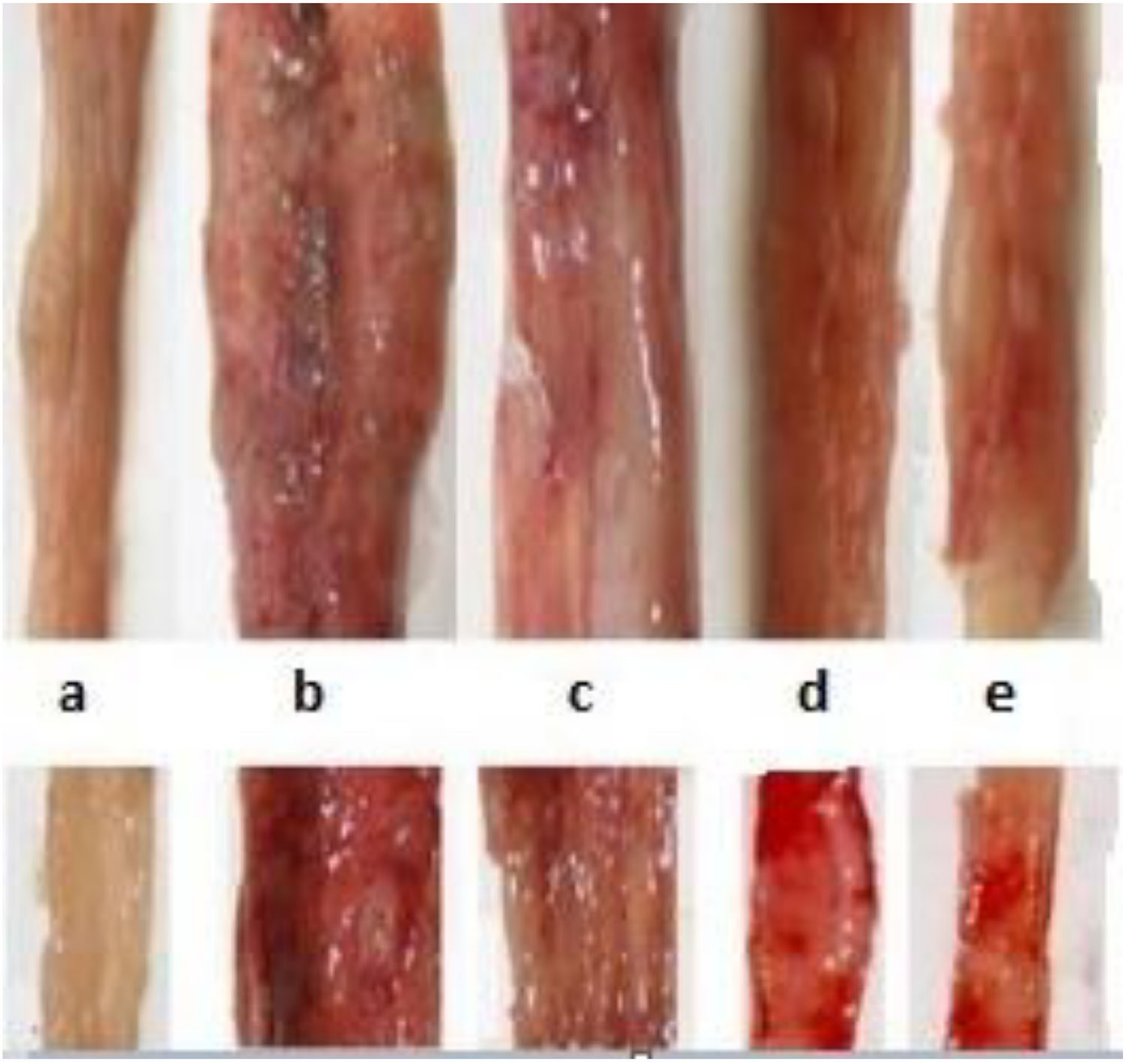
Representative images of macroscopic changes in colonic tissue across experimental groups. (a) Control; (b) ulcerative colitis; (c) mesalazine; (d) *Bacillus coagulans*; (e) *Lactobacillus acidophilus*; each intestinal segment measures approximately 4 cm in length. The control group tissue showed a normal appearance with no signs of ulceration, hyperaemia, inflammation, or oedema. In contrast, the ulcerative colitis group (b) exhibited severe tissue injury, with darkened areas indicating extensive ulceration, hyperaemia, inflammation, and oedema. Treatment with mesalazine or probiotics (c–e) markedly reduced macroscopic damage, with no evidence of ulceration and a clear reduction in hyperaemia, inflammation, and oedema compared with the ulcerative colitis group. *n* = 8 mice per group.

**FIGURE 4:**
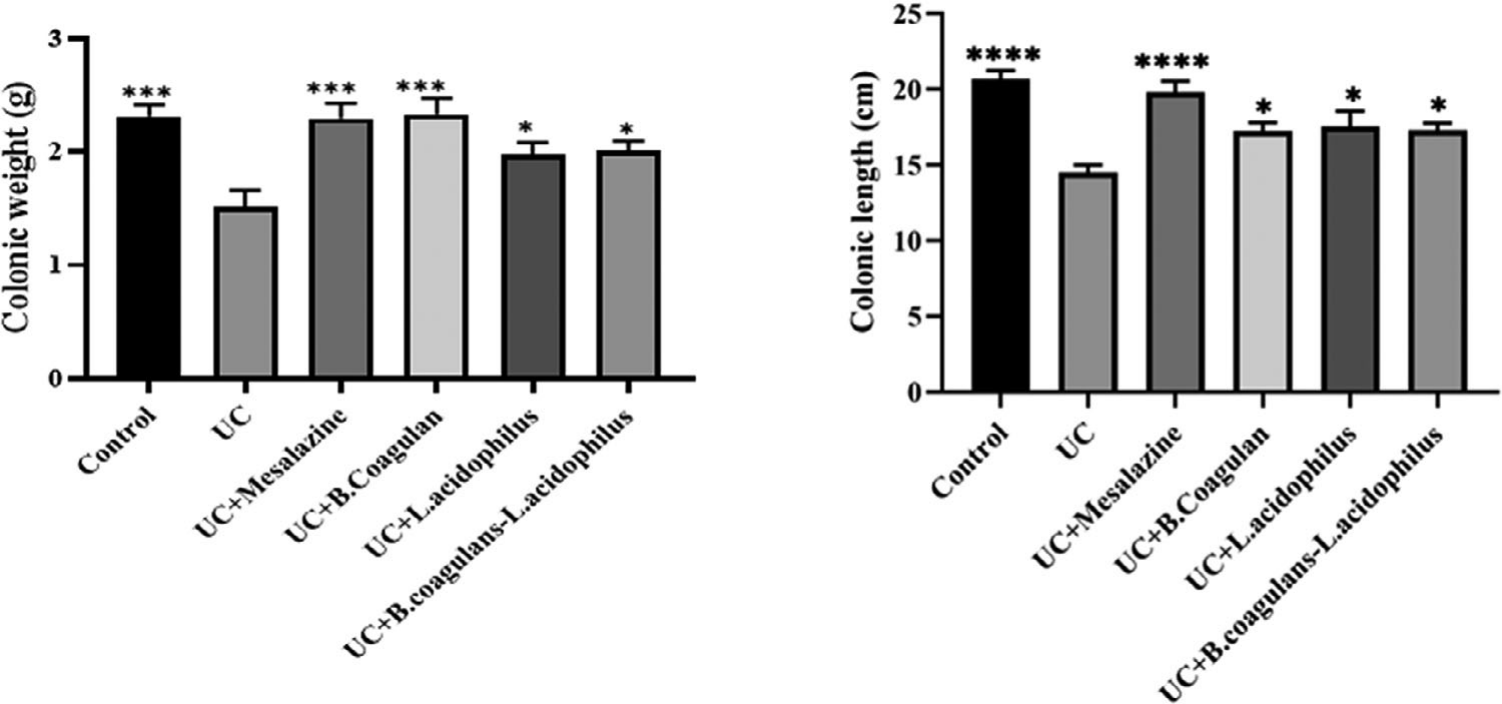
Effects of mesalazine, *Lactobacillus acid*Effects of mesalazine, Lactobacillus acidophilus, Bacillus coagulans on colon length and weight in acetic acid–induced ulcerative colitis. Measurements were performed on colonic tissue, and data are presented as mean ± SEM. ****p < 0.0001 vs. control group; *p < 0.01, ***p < 0.0001 vs. ulcerative colitis group. *n* = 8 mice per group.

### 3.4 Colon Length and Weight Analysis

Macroscopic indices, including colon length and weight, were used as measures of tissue oedema. The induction of colitis led to a significant decrease in colon length (FC = 6.188, *p* < 0.0001) and colon weight (FC = 0.7925, *p* < 0.0001) compared with the healthy control group. Treatment with mesalazine (300 mg/kg) significantly restored colon length (FC = −5.313, *p* < 0.0001) and colon weight (FC = −0.7800, *p* = 0.0002). Similarly, the administration of *L. acidophilus* (FC = −3.063, *p* = 0.0113; FC = −0.4575, *p* = 0.0442) and *B. coagulans* (FC = −2.750, *p* = 0.0481; FC = −0.8142, *p* = 0.0003) significantly improved both parameters compared with the ulcerative colitis group. These findings indicate that probiotic interventions, similar to mesalazine, effectively attenuate tissue oedema in colitis (Figure 4).

### 3.5 Histopathological Evaluation of Colonic Tissue in Experimental Ulcerative Colitis

Histological analysis of colon sections stained with H&E (×10 magnification) demonstrated marked differences between the experimental groups. In the ulcerative colitis group (Figure 5c), a significant increase in the thickness of the mucosa, submucosa, and muscularis layers was observed, indicating severe oedema and inflammation compared to the control group. In contrast, samples treated with mesalazine (Figure 5a) and probiotics (*B. coagulans* and *L. acidophilus*; Figure 5d,e) showed a notable reduction in the thickness of these layers, suggesting the attenuation of oedema and inflammatory responses under treatment. The purple‐stained cells represented inflammatory cell infiltration, including neutrophils and lymphocytes. A high density of these cells was evident in the ulcerative colitis group (Figure 5c), reflecting severe inflammation, neutrophil infiltration, lymphocyte infiltration, and lymphoid hyperplasia across all tissue layers. However, mesalazine and probiotic treatments markedly reduced the number of inflammatory cells in the mucosa, submucosa, and muscularis, confirming their anti‐inflammatory effects compared with the ulcerative colitis group (Figure 5d,e).

**FIGURE 5:**
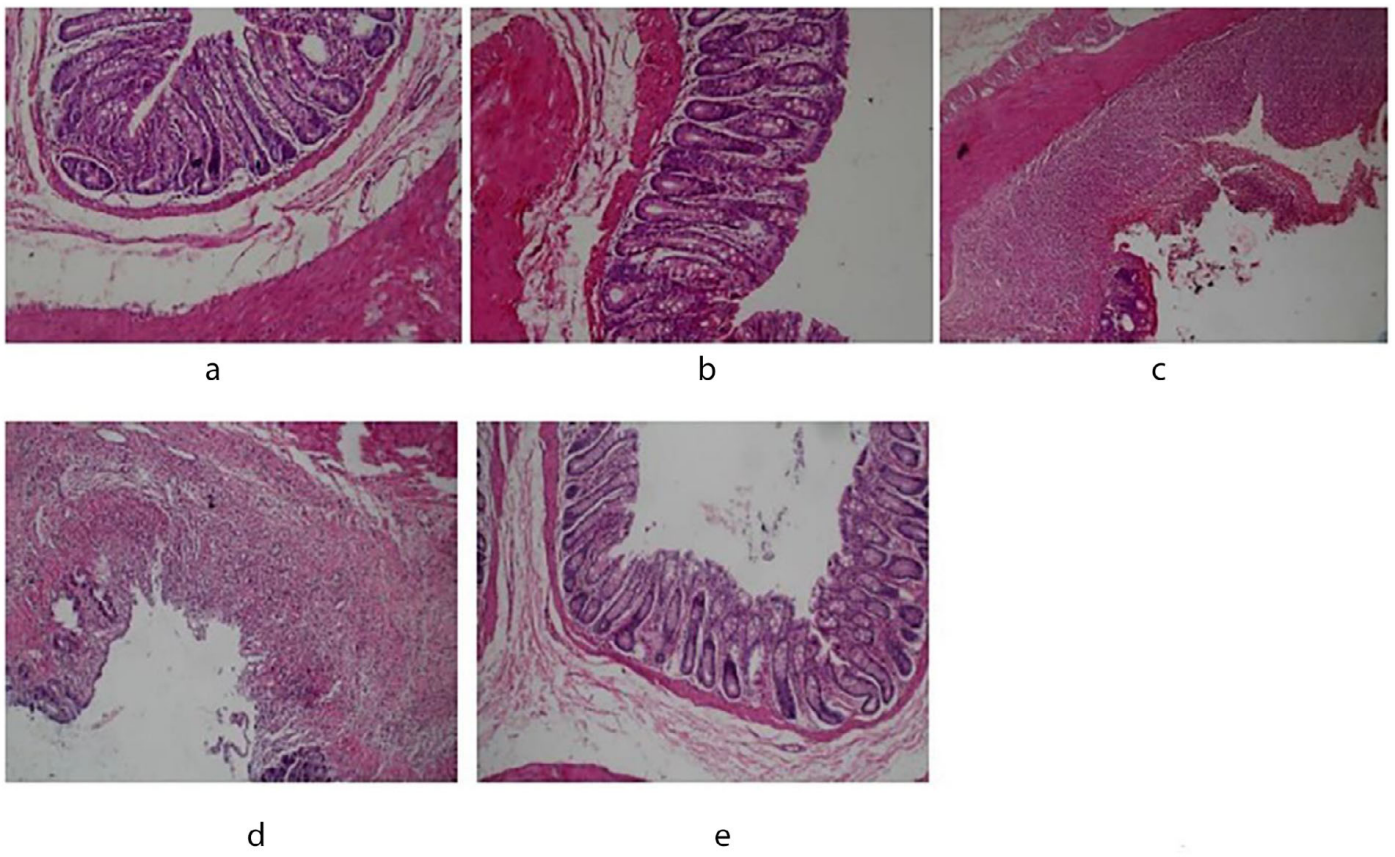
Histopathological changes in colon tissue across experimental groups. Representative H&E‐stained sections are shown for (a) mesalazine‐treated group (×40), (b) healthy control (×10), (c) ulcerative colitis group (×4), (d) *Bacillus coagulans* probiotic (×4), and (e) *Lactobacillus acidophilus* probiotic (×10).

### 3.6 Evaluation of Macroscopic and Histopathological Scoring in Colon Tissues

Table 3 summarizes the macroscopic and histopathological scores of colon tissues across the experimental groups. The ulcerative colitis group showed the highest macroscopic wound score (mean: 3.25) accompanied by severe inflammation, moderate crypt damage, and up to 75% tissue involvement, indicating extensive ulceration and colonic injury. In contrast, the control group displayed normal architecture with intact crypts, no inflammatory infiltration, and absence of macroscopic lesions.

Treatment with mesalazine, *B. coagulans*, and *L. acidophilus* significantly reduced both macroscopic and microscopic damages compared to the ulcerative colitis group. These groups demonstrated marked improvement in mucosal integrity, reduced inflammatory cell infiltration, and lower engagement percentages, reflecting a protective effect against colitis‐associated injury. Among the probiotic‐treated groups, the *B. coagulans* and *L. acidophilus* showed superior outcomes, comparable to mesalazine, in ameliorating both macroscopic lesions and histopathological alterations (Table 4).

**TABLE 4:** Scoring of macroscopic and histopathological changes in colon tissue across experimental groups.

Groups	Mean macroscopic wound score	Severity of inflammation	Extent of inflammation	Crypt damage	Engagement percentage	Histopathological score
Control	0	No inflammation	0	Intact crypts	0	0
Ulcerative colitis	3.25	Severe	3	Moderate	51–75	4
Mesalazine	1.50	Mild	1	Mild	1–25	2
*Bacillus coagulans*	1.83	Moderate	2	Mild	1–25	2
*Lactobacillus acidophilus*	1.50	Mild	1	Mild	1–25	2

Microscopic analysis of colon tissue in the colitis group revealed pronounced pathological changes, including extensive epithelial cell loss, a marked reduction in goblet cells, severe leukocyte infiltration, and thickening of the muscular layer (Figure 6). As shown in Table 4, the ulcerative colitis group exhibited the highest macroscopic and histopathological damage scores. In contrast, treatment with mesalazine and probiotic formulations led to a significant reduction in tissue injury and inflammatory responses compared to the colitis group.

**FIGURE 6:**
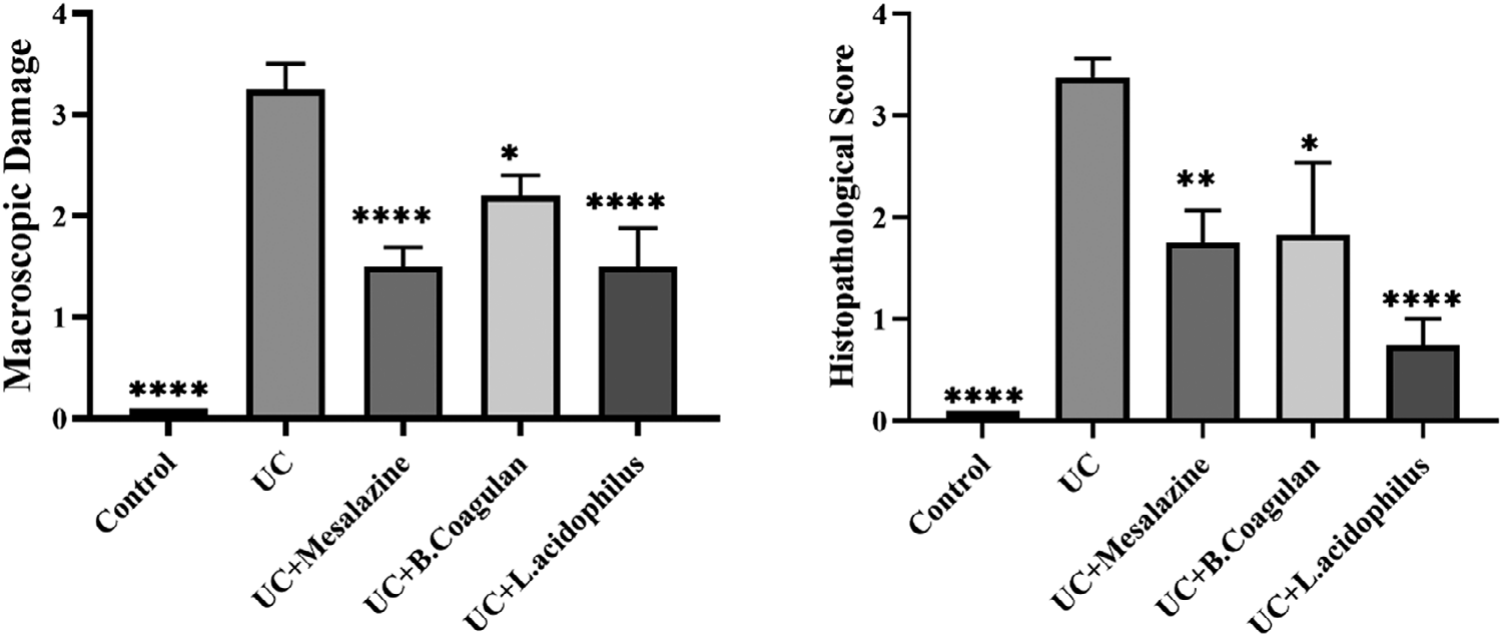
Effects of mesalazine and the probiotics Lactobacillus acidophilus and Bacillus coagulans on macroscopic and histopathological lesions in acetic acid‐induced ulcerative colitis. Data are presented as mean ± SEM. *p < 0.05, **p < 0.01, ****p < 0.0001 versus the ulcerative colitis group. Each group included *n* = 8 mice.

## 4 Discussion

The present study investigated the therapeutic effects of *B. coagulans* and *L. acidophilus*, individually and in combination, on acetic acid–induced ulcerative colitis in a small animal model. Our findings demonstrate that probiotic interventions significantly reduced macroscopic and microscopic lesions, downregulated inflammasome‐associated genes, and attenuated pro‐inflammatory cytokine levels. These results highlight the potential of probiotics as complementary or alternative strategies to conventional therapies, such as mesalazine in veterinary medicine (Allenspach 2015; Bakhshinejad et al. 2014).

Inflammatory bowel diseases in animals share many pathological features with human colitis, including dysregulated immune responses and impaired epithelial barrier integrity (Washabau and Day 2013). In veterinary practice, the treatment of chronic colitis often relies on immunosuppressive agents, antibiotics, or corticosteroids, which are associated with significant side effects and treatment failures (Dandrieux 2016). Hence, safer alternatives, such as probiotics, are highly desirable. Our data provide experimental evidence supporting the integration of probiotics into veterinary gastroenterology.

One of the central findings was the marked downregulation of NLRP3, Caspase‐1, IL‐18, and TNF‐α gene expression in probiotic‐treated groups. The dysregulation of inflammasome signalling has been implicated in persistent mucosal inflammation in both human and veterinary colitis (Lamkanfi and Dixit 2014). By reducing inflammasome activation, probiotics likely interrupt a key pathway driving tissue injury. These observations align with earlier studies in murine and canine colitis, showing that probiotic supplementation attenuates inflammasome‐mediated responses (Zhen and Zhang 2019).

Probiotics not only modulated gene expression but also significantly reduced IL‐1β and IL‐17 protein levels in colon tissue. Both cytokines are central drivers of neutrophil recruitment, lymphocyte activation, and mucosal barrier disruption (Jergens and Simpson 2012; Khademi et al. 2026). Their reduction in treated groups indicates that probiotics effectively suppress downstream inflammatory cascades. This is consistent with previous findings in dogs supplemented with *Lactobacillus* strains, where reductions in pro‐inflammatory cytokines correlated with clinical improvement (Schmitz and Suchodolski 2016; Herstad et al. 2022).

Macroscopic indices, such as colon length and weight, revealed that probiotic treatment restored tissue morphology and reduced oedema. These findings mirror earlier veterinary studies showing that probiotics can prevent mucosal shortening and swelling in colitis models (Wirtz et al. 2017; Rodríguez‐Padilla et al. 2021). The restoration of tissue morphology is clinically significant, as it reflects improved epithelial healing and reduced inflammation, which are critical for long‐term disease control in animals.

Histopathological evaluations confirmed that probiotics markedly attenuated mucosal and submucosal damage, reduced crypt destruction, and lowered inflammatory cell infiltration. The protective effects observed were comparable to mesalazine, a standard treatment for colitis (Jergens et al. 2010). The reduction of crypt abscesses and the restoration of goblet cells in probiotic groups support their role in enhancing epithelial repair, consistent with reports in calves and dogs receiving *Lactobacillus*‐based formulations (Uyeno et al. 2015).

Interestingly, both *B. coagulans* and *L. acidophilus* demonstrated individual efficacy, but their combined use showed broader anti‐inflammatory benefits. Multi‐strain probiotic formulations are thought to exert synergistic effects by targeting different aspects of gut ecology and immune regulation (Chapman et al. 2011a, 2011b). The enhanced efficacy of the probiotic mixture in this study supports previous veterinary research, where multi‐strain formulations showed superior outcomes compared with single strains (Timmerman et al. 2004).

The resilience of *B. coagulans* spores to harsh gastrointestinal conditions offers practical advantages for veterinary applications, especially in farm animals where environmental conditions vary (Arzi et al. 2018). Meanwhile, *L. acidophilus* exhibits strong colonization and immune‐modulating properties, making it suitable for companion animals with chronic enteropathies (Ouwehand, Salminen, and Isolauri 2002). Their complementary features may explain the observed synergistic benefits.

Our results also suggest that probiotic interventions can reduce reliance on pharmacological therapies. such as mesalazine. Although mesalazine effectively reduced inflammation, long‐term use in animals may be limited by cost and potential side effects (Dandrieux 2016). Probiotics provide a safer and potentially more sustainable option, particularly in livestock, where minimizing drug residues is critical for food safety (Uyeno et al. 2015).

The relevance of probiotics extends beyond colitis therapy to broader veterinary health management. By modulating microbiota composition, probiotics can enhance resistance against enteric pathogens; reduce diarrhoea in calves, piglets, and poultry; and improve nutrient absorption (Hun 2020a, 2020b). Thus, the findings of this study contribute not only to colitis management but also to improving overall animal health and productivity.

Mechanistically, the suppression of inflammasome signalling by probiotics may occur via modulation of Toll‐like receptor pathways, production of short‐chain fatty acids, and enhancement of epithelial integrity (Bermudez‐Brito et al. 2012). These pathways have been implicated in both murine and canine colitis models, supporting the translational value of our findings (Li et al. 2021).

Despite these promising results, some inflammation and leukocyte infiltration persisted in the probiotic‐treated groups. This suggests that while probiotics alleviate disease severity, they may not completely replace standard pharmacological therapy in severe cases. Instead, probiotics could serve as adjuncts, enhancing therapeutic efficacy while reducing drug doses and associated side effects (Schmitz and Suchodolski 2016).

The translational value of small animal models for veterinary medicine must also be emphasized. Murine models provide controlled settings to test interventions, but differences in microbiota composition and immune function between rodents and target veterinary species should be acknowledged (Wirtz et al. 2017). Therefore, clinical trials in dogs, cats, and livestock are necessary to confirm the therapeutic potential of *B. coagulans* and *L. acidophilus*.

## 5 Conclusion

In conclusion, our findings demonstrate that *B. coagulans* and *L. acidophilus*, individually and in combination, reduce inflammasome activation, attenuate pro‐inflammatory cytokines, and ameliorate colonic lesions in experimental ulcerative colitis. These results support the potential of probiotics as safe, effective adjunct therapies for colitis in veterinary medicine. Future research should focus on clinical trials in veterinary species, optimization of dosing regimens, and formulation of multi‐strain probiotic products tailored for different animal populations.

## Author Contributions

**Fatemeh Shayesteh**: conceptualization, methodology, formal analysis, investigation, writing – original draft, and writing – review and editing. Milad Shahini Shams Abadi: methodology and investigation. **Leila** Rouhi: methodology and investigation. **Abbas Doosti** and **Nader Bagheri** conceptualization and funding acquisition. All authors have read and approved the final version of the manuscript.

## Funding

The authors have nothing to report.

## Ethics Statement

This article is part of the research of a PhD student in microbiology with the ethics code IR.IAU.SHK.REC.1404.078.

## Conflicts of Interest

The authors declare no conflicts of interest.

## Data Availability

The data that support the findings of this study are available from the corresponding author upon reasonable request.
